# Advances in the Study of the Pathogenesis of Vogt-Koyanagi-Harada Syndrome

**DOI:** 10.2174/0115665240311578241014050805

**Published:** 2024-10-24

**Authors:** Meng-nan Wang, Lin Zhang, Hong-Yan Zhou, Wei Zhong, Hong Zhang, Miao-Miao Bi, Li Wang, Jia Lu

**Affiliations:** 1 Department of Ophthalmology, China-Japan Union Hospital, Jilin University, Changchun, Jilin 130033, P.R. China

**Keywords:** Autoimmune disease, pathogenesis, Vogt-Koyanagi-Harada Syndrome

## Abstract

Vogt-Koyanagi-Harada syndrome (VKHS) is a common type of uveitis characterized by the invasion of melanocyte-rich tissues. In recent years, the incidence of VKHS has been increasing yearly, and its specific pathogenesis has not yet been elucidated. However, its pathogenesis has been a hot topic of research. The clinical course of VKHS is characterized by the early involvement of the posterior segment of the eye, including exudative retinal detachment, optic papillitis, bilateral diffuse chorioretinitis, etc. If treated improperly or with delayed treatment, the inflammation may gradually spread to the anterior segment of the eye, leading to vision loss or even vision. This study examines the pathogenesis of VKHS. It reviews the progress of research on the pathogenesis of VKHS, which will help to improve the understanding of VKHS and provide a reference for subsequent studies.

## 1 INTRODUCTION

Vogt-Koyanagi-Harada Syndrome (VKHS) is one of the common types of uveitis, also known as “idiopathic uveitis” and “oculo-cerebral-ear-skin syndrome”. VKHS is a multisystem autoimmune disease characterized by non-granulomatous uveitis in both eyes, usually accompanied by involvement of the skin, hair, auditory system, and central nervous system. VKHS is characterized by a high recurrence rate, rapid onset, rapid disease progression, and a high rate of blindness. VKHS is a common type of endogenous total uveitis that affects patients' quality of life and visual quality due to its poor visual acuity and ocular complications [1]. Appropriate therapeutic measures for patients with VKHS can not only effectively improve their visual function but also significantly enhance their psychological status and overall quality of life [2]. VKHS is widespread worldwide and occurs in people with darker skin, especially those of Asian, Middle Eastern, North American, and Spanish descent [3]. Up to now, the pathogenesis of VKHS reported in the national and international literature involves a complex immunologic process, a specific genetic background, and possible infectious factors [4]. VKHS is essentially a stromal chorioretinitis, with typical histopathologic features of lymphocytes, epithelioid cells, histiocytes, and multinucleated giant cells in the middle and outer tissues of the choroid as the main manifestation of extensive inflammation. The treatment of VKHS relies mainly on glucocorticoids, immunosuppressants, and biological agents. These drugs are effective in suppressing abnormal immune system responses and reducing inflammation, thereby protecting vision and improving patients' quality of life.

## 2 PATHOGENESIS

### 2.1 Immunopathogenesis

#### 2.1.1 T lymphocytes

The increase in CD4+ T cell subsets can be detected in the cerebrospinal fluid and aqueous humor of VKHS patients [5]. CD4+ T cells can be induced to differentiate into Th17 cells, whereas Interleukin (IL)-23 promotes the secretion of IL-17 from Th17 cells. IL-23 is primarily responsible for the ongoing activity of inflammation, inducing IL-17 production and CD4+ T cell subpopulation differentiation, and plays a key role in the development and maintenance of autoimmune inflammation [6, 7]. Recent studies have reported the important role of IL-23 in inducing the differentiation of Th17 cells, the major CD4+ T cell subset that produces IL-17. This finding has important implications for understanding the onset and progression of VKHS. Abnormal activation of the IL-23/IL-17 pathway was found to play a key role in the course of VKH. IL-17 produced by Th17 cells promotes the release of inflammatory mediators, which leads to an increased inflammatory response, which, in turn, triggers tissue damage. IL-23-mediated activation of Th17 cells may promote the development of autoimmune responses, leading to the emergence and progression of symptoms in patients with VKHS. The findings suggest that the inflammatory response in VKH may be associated with the upregulation of IL-23 and IL-17 expression [7].

IL-35 belongs to a new member of the IL-12 family [8]. IL-35 is a cytokine produced by natural regulatory T cells, which not only inhibits the proliferation and differentiation of Th17 cells but also promotes the function of regulatory T cells (Tregs) [9]. IL-35 plays an important role in a variety of autoimmune disorders, such as Behcet’s disease [10] and Sjogren’s syndrome [11, 12]. In patients with active VKHS, the expression level of IL-35 is significantly reduced. IL-35 has an immunosuppressive function to maintain Treg cells, and its reduced expression leads to a weakening of the inhibitory ability of Treg cells to suppress inflammatory responses, which, in turn, triggers or exacerbates inflammatory responses [13].

**Fig. (1):**
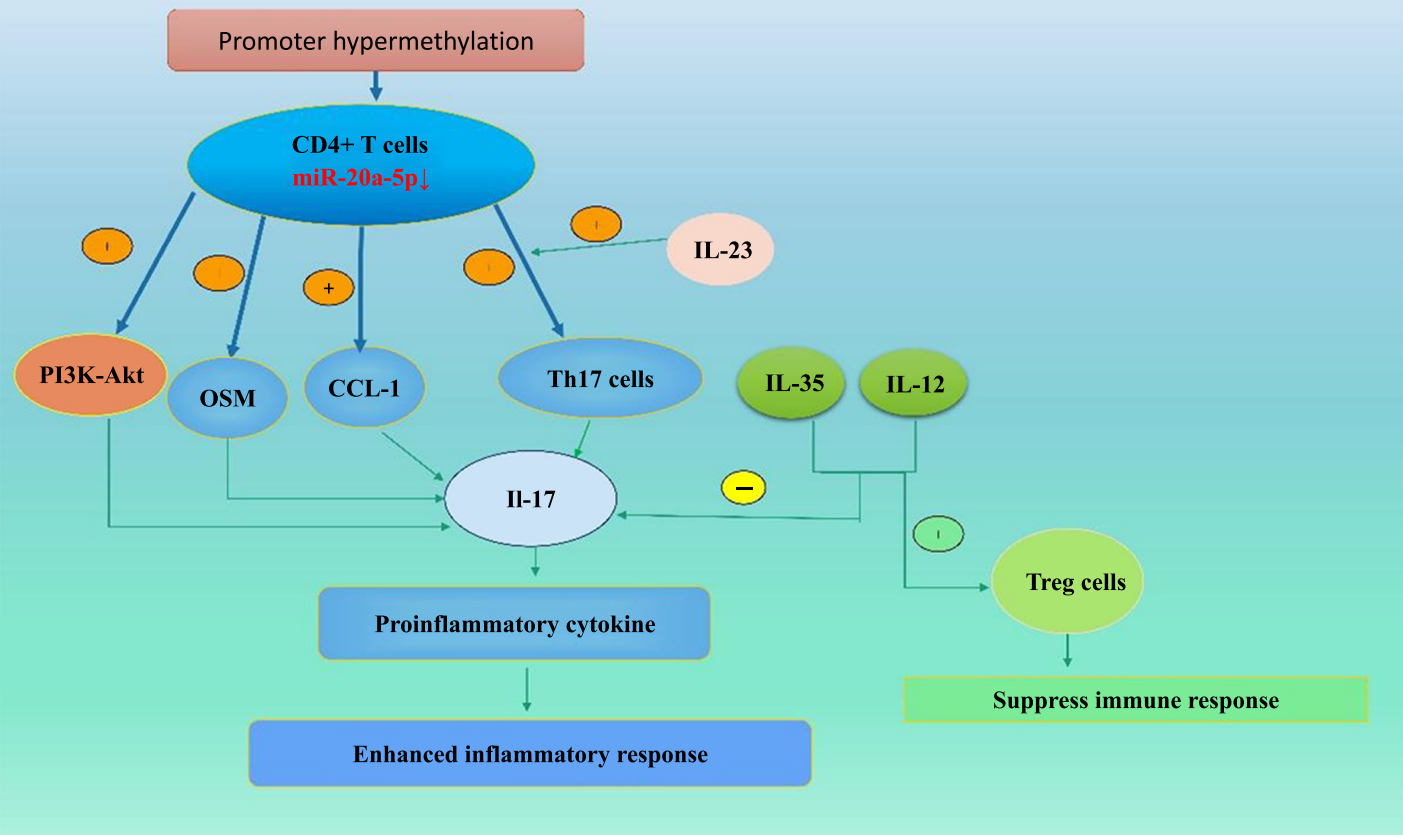
The process of enhanced inflammation resulting from the significant reduction of miR-20a-5p in CD4+ T lymphocytes *in vivo* in patients with active VKHS.

**Fig. (2):**
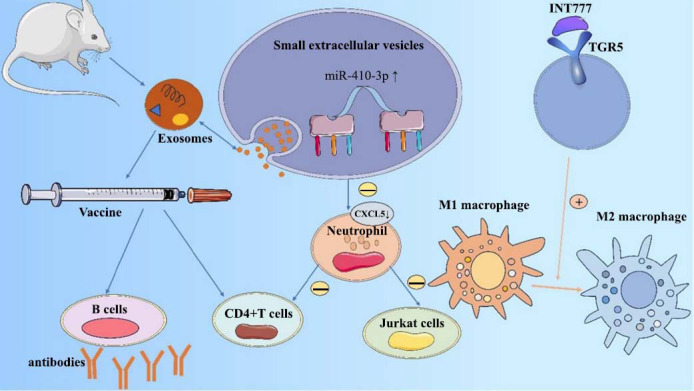
Small extracellular vesicles inhibit the proliferation of CD4+ T cells and Jurkat cells by regulating CXCL5 through miR-410-3p. The isolated exosomes extracted from rats were inoculated with a vaccine, which stimulated the body's B cells to secrete antibodies and the CD4+ T cells to undergo an immune response, thereby inducing the development of immune tolerance.

It has been shown that the expression levels of certain specific miRNAs are significantly altered in patients with active VKH and that these changes affect the immune response of T cells, which, in turn, are involved in the pathogenesis of VKHS. The study by Chang *et al*. [14] demonstrated that microRNA-20a-5p (miR-20a-5p) expression levels in CD4+ T cells were significantly reduced in patients with active VKHS, along with an increased frequency of promoter methylation of miR-20a-5p. This suggests that miR-20a-5p expression may be inhibited by promoter methylation. miR-20a-5p targets oncoprotein M (OSM) and C-C motif chemokine ligand 1 (CCL1). It is a microRNA that regulates gene expression at the post-transcriptional level. OSM and CCL1 are two known target genes of miR-20a-5p. Studies have reported that miR-20a-5p is able to inhibit IL-17 production in CD4+ T cells from patients with active VKHS. The miR-20a-5p has also been found to negatively regulate the phosphatidylinositol 3-kinase (PI3K)-Akt signaling pathway. Moreover, it inhibits this pathway by targeting and inhibiting key components in the PI3K-Akt signaling pathway, such as the catalytic subunit of PI3K or the Akt proteins, to inhibit the activity of the pathway. This helps to reduce IL-17 production and inflammatory responses. Furthermore, miR-20a-5p downregulation may affect CD4+ T cell function, which, in turn, leads to an overreaction of the immune system to self-antigens and promotes inflammation and tissue damage. These findings suggest that new therapeutic approaches could be developed to modulate immune responses in the future based on the mechanism of interaction between miR-20a-5p, OSM, and CCL1 and that changes in the levels of miR-20a-5p may be an important biomarker for the diagnosis and monitoring of VKHS [14] (Fig. **1**). Small extracellular vesicles (sEVs) are a class of small vesicles released by cells and isolated from their surroundings by a lipid bilayer, with diameters ranging from 30 nm to 1000 nm [15]. The sEVs include exosomes, microvesicles, and apoptotic vesicles. Exosomes are nanosized vesicles secreted by a variety of cell types, and they are typically between 30 and 150 nm in diameter. These tiny vesicles contain a variety of biomolecules, such as RNAs (both mRNAs and non-coding RNAs, such as miRNAs), proteins, and lipids. Exosomes play an important role in intercellular communication, delivering signaling molecules, regulating gene expression, and participating in a variety of biological processes, such as immune regulation and tumor progression [16]. The levels of miR-410-3p are significantly upregulated in circulating sEVs during the active phase of uveitis in VKH patients. miR-410-3p is able to inhibit the proliferation of Jurkat cells (a T-lymphocyte cell lineage) and CD4+ T-cells, implying that miR-410-3p affects the immune response by inhibiting the activity of these immune cells. These data suggest that miR-410-3p could be used as a biomarker for the early diagnosis of VKH or for monitoring disease activity [17] (Fig. **2**). The use of exosomes isolated from the plasma of rats with autoimmune uveitis as a vaccine component is an approach that not only reduces the severity of the disease but also opens new avenues for the treatment of autoimmune uveitis [18].

It was found that the mRNA and protein expression levels of DAB2 were higher in dendritic cells of normal controls and quiescent VKHS patients than in active VKHS patients. This suggests that the low expression of DAB2 in patients with active VKHS may contribute to the triggering or exacerbation of the body's autoimmune response [19].

#### 2.1.2 B Lymphocytes

B lymphocytes are able to differentiate into plasma cells and produce antibodies. In VKHS, these antibodies may target eye-specific antigens, triggering an immune response and tissue damage. In addition to producing antibodies, B lymphocytes secrete a variety of cytokines. These cytokines are involved in the regulation of the immune response and may directly or indirectly contribute to the onset and progression of the inflammatory response [20, 21]. Moreover, therapeutic approaches targeting B lymphocytes prove effective in treating VKHS [22]. The levels of CXCL13 in aqueous humor samples from patients with VKHS are significantly higher than those from patients with leukodystrophy Behcet’s disease (BD) and patients with human leukocyte antigen (HLA)-B27-associated uveitis [23, 24]. CXCL13 acts as a chemokine that directs the movement of B lymphocytes to peripheral immune organs [25]. This migration is essential for initiating and maintaining the immune response.

#### 2.1.3 Macrophages

Macrophages play an important role in antigen presentation and inflammatory regulation. Two different types of macrophages have different physiological functions; M1 macrophages have the ability to release pro-inflammatory cytokines, while M2 macrophages have the opposite role [26, 27]. G protein-coupled bile acid receptor 1/Gpbar1 (also known as TGR5) is a cell membrane receptor that can be activated by recognition of semisynthetic bile acid INT-777, which prompts the M1 macrophage to M2 macrophage differentiation. It was found that there were significantly more TGR5 in M1 macrophages in normal controls than in patients with active VKHS, implying that TGR5 expression may be decreased during the active phase of VKHS. Since the TGR5 receptor promotes the conversion of M1 macrophages to M2 macrophages, decreased TGR5 expression may hinder this process, leading to a relative increase in the proportion of M1 macrophages and the release of more pro-inflammatory cytokines, which further exacerbates the inflammatory response [28].

#### 2.1.4 Abnormal Immune Regulation

Almost all self-reactive lymphocytes from VKHS patients express Fas antigen (Fas Ag, a membrane protein associated with apoptosis) [29]. When Fas binds to its ligand Fas ligand (FasL), it triggers a series of signaling pathways that ultimately lead to apoptosis. Fas Ag has a strong cytotoxic effect upon binding to FasL, causing not only apoptosis of T lymphocytes expressing Fas Ag but also paracrine death of neighboring T lymphocytes. Thus, the imbalance of the Fas/FasL system may be one of the important causes of chronicity and recurrent episodes of VKHS. Recent research evidence suggested that both Fas Ag and Bcl-2 are strongly expressed on CD4+ T cells in aqueous humor and cerebrospinal fluid samples from VKHS patients and that Bcl-2 plays the opposite role to Fas Ag, which inhibits apoptosis. Due to the presence of Bcl-2, autoreactive T cells may evade apoptosis even in the presence of FasAg upregulation, leading to their accumulation in the body and continued triggering of an autoimmune response, leading to the chronicity of the disease.

#### 2.1.5 Autoimmunity

VKHS is considered an autoimmune disease characterized by an abnormal immune response of the body to melanocytes or melanocyte-related antigens [30]. This immune response is not limited to ocular tissues but may also spread to other melanocyte-rich sites, such as the hair, inner ear, and nervous system [30]. T lymphocyte-targeting proteins in patients with VKHS include tyrosinase, tyrosine-associated protein 1, tyrosine-associated protein 2, and gp100 [31, 32].

### 2.2 Genetic Pathogenesis

#### 2.2.1 Macrogenetics

Genetic studies on VKH do suggest that the disease is associated with certain genetic factors. These genetic factors can be broadly categorized into two main groups: HLA-related factors and non-HLA factors. HLA, also known as the major human histocompatibility antigen, is associated with a variety of autoimmune diseases, such as rheumatoid arthritis [33], ankylosing spondylitis [34], and Hashimoto's thyroiditis [35]. Several studies have confirmed that certain specific alleles in the HLA system (*e.g*., HLA-DR4, HLA-DRw53, and especially HLA-DRB1*0405) are strongly associated with the development of VKHS [36-39]. Carriers of these alleles have a significantly higher risk of developing the disease than non-carriers. Since HLA-DQB1*0405 recognizes more melanocyte-associated peptides, this means that the immune system may be more easily activated, which, in turn, may lead to a more complex immune response [40].

A meta-analysis of patients with VKHS concluded that specific HLA-DQA1 and HLA-DQB1 alleles may have a protective effect, reducing an individual's risk of developing VKHS, and these findings also emphasize the positive correlation between the HLA-DQA1*0301 and HLA-DQB1*0401 alleles and the risk of developing VKHS, *i.e*., individuals carrying these alleles have a higher risk of developing VKHS [41].

There are relatively few studies in the literature on the relationship between non-HLA factors and VHKS. Du *et al*. [42] found genetic polymorphisms of cytotoxic T lymphocyte-associated antigen-4 to be associated with susceptibility to VKHS in a Chinese population. In addition, findings showed that single nucleotide polymorphisms +49 of the G allele and haplotype -1661A:-318C:+49G: CT60G were associated with the risk of developing VKHS [42].

#### 2.2.2 Epigenetics

Epigenetics refers to changes that do not involve gene sequence changes but altered gene expression, including DNA methylation, histone modifications, and the role of noncoding RNAs. The association between epigenetic inheritance and VKHS has been noted in recent studies. Interferon regulatory factor 8, GATA-binding protein 3, IL-4, and transforming growth factor-β promoter methylation levels are significantly up-regulated in patients with active VKHS, which affects the regulatory function of the immune cells and thus is associated with an abnormal inflammatory response in VKHS [43, 44]. Studies have identified an association between VKHS and specific miRNA copy number variants. The miRNAs are a class of short-stranded non-coding RNA molecules that regulate gene expression by binding to the 3' untranslated region (3'UTR) of target mRNAs. This regulation is essential for maintaining normal physiological functions and plays an important role in disease states. In VKHS patients, copy number variants of miR-23a, miR-146a, and miR-301a may alter the expression of their target genes, which, in turn, affects the function of immune cells [45].

### 2.3 Abnormal Amino Acid Metabolic Pathways

Indoleamine 2,3-dioxygenase 1 (IDO1) is a rate-limiting enzyme of tryptophan catabolism responsible for catalyzing the first step of tryptophan metabolism, converting it to kynurenine. IDO1 exhibits high expression in a wide range of immune cells of active VKHS. Tryptophan is one of the essential amino acids for T cell proliferation, and IDO1 inhibits T cell proliferation by degrading tryptophan and promotes the differentiation of CD4+ T cells toward Treg cells [46]. In fact, IDO-catalyzed increases in tryptophan metabolism have been shown to be associated with many human autoimmune diseases, including systemic lupus erythematosus [47], rheumatoid arthritis [48], and inflammatory bowel disease [49]. It has been found that IDO1 suppresses the intrinsic and adaptive immune responses through increased kynurenine production and decreased tryptophan, thereby inducing peripheral immune tolerance [50].

Cui *et al*. [51] found that metabolomic analysis of sweat from VKHS patients showed lower levels of L-serine, 1-phosphoglycerate dehydrogenase (PHGDH), L-tryptophan, choline, and betaine than in healthy controls. The enzyme PHGDH plays a key role in the conversion of 3-phosphoglycerate to L-serine. L-serine deficiency may lead to cell death and stress response, which may be part of the pathogenesis of VKHS [52]. Excessive reactive oxygen species (ROS) have direct toxic effects on tissue cells. L-tryptophan plays an important role in scavenging ROS and regulating the body's immune response [53, 54]. Therefore, changes in L-tryptophan levels may affect the immune response in patients with VKHS. Choline and betaine are involved in the synthesis of trimethyl N-oxide. Deficiency of these two substances promotes the production of ROS, which, in turn, leads to increased oxidative stress and inflammatory responses [55, 56].

Some studies have reported that autoimmune diseases are associated with amino acid-tRNA biosynthesis [57-59]. Xu *et al*. [60] showed that the biosynthesis of pantothenic acid and coenzyme A was altered in the aqueous humor of VKHS patients. Pantothenic acid and coenzyme A are important metabolites, and their changes may reflect abnormal metabolic processes in VKHS patients. In addition, it was found that both VKHS and Behcet’s disease (BD) patients had increased L-histidine and L-lysine, suggesting a possible involvement of amino acid-tRNA biosynthetic pathways in their pathogenesis [60]. Since reduced levels of 1,25-(OH)2-D3, the active form of vitamin D, can significantly enhance the release of inflammatory cytokines, it was found that serum 1,25-(OH)2-D3 levels were significantly lower in patients with active VKHS than in normal controls and patients with quiescent VKHS [61].

### 2.4 Cellular and Molecular Mechanisms

A study used mRNA sequencing technology to analyze peripheral blood mononuclear cells (PBMCs) in VKHS patients. By comparing mRNA expression in PBMCs from patients with active VKHS before treatment, after treatment, and in healthy controls, the results showed that there were differences in the mRNA expression profiles between the groups, and the study revealed that the peripheral immune cells were not only involved in the pathogenesis of VKHS but that the changes in their status also reflect the patient's response to treatment. The study further revealed the underlying cellular and molecular mechanisms of VKHS, particularly emphasizing the important role of monocytes in the pathogenesis of VKHS and the importance of tribble 1 (TRIB1) as a potential biomarker for monitoring disease progression and response to therapy. The study showed that in patients with active uveitis in VKHS, TRIB1 levels tended to increase and then decrease before and after treatment. This suggests that changes in TRIB1 may be associated with disease activity and treatment efficacy [62]. The study reveals the roles of two key signaling pathways, the arachidonic acid metabolic pathway and the mitogen-activated protease (MAPK) signaling pathway, in the pathogenesis and treatment of VKHS. The study suggests that the arachidonic acid metabolic pathway may be associated with the inflammatory response in VKHS. The MAPK signaling pathway may be one of the potential targets for VKHS treatment. Both prednisone and cyclosporine A can attenuate the inflammatory response and immune response by modulating the MAPK signaling pathway, thus achieving therapeutic objectives [63].

### 2.5 Infection Pathogenesis

Although the exact etiology is not clear, some studies have suggested that viral infection may be a factor in triggering VKHS. The research reveals the possible role of viral infections in triggering autoimmune diseases, such as VKH disease, through molecular mimicry mechanisms. Several studies have suggested that viral infections, including hepatitis B virus [64], hepatitis C virus [65], and cytomegalovirus (CMV) [66], may trigger immune responses to tyrosinase-associated peptides through molecular mimicry mechanisms, which may in turn induce VKH disease. Sugita *et al*. [31] found that due to tyrosinase, CMV-related peptides have amino acid sequences with high homology. Molecular mimetic mechanisms play an important role in the pathogenesis of VKHS. After CMV infection, T cells not only generate an immune response to the virus but may also mistakenly attack tyrosinase, which has structural similarity to the viral antigen, thereby affecting melanin synthesis [66-68].

Gut microbes are involved in several autoimmune diseases, such as multiple sclerosis [69, 70], Behcet’s disease [71], and systemic lupus erythematosus [72]. In recent years, studies on the relationship between VKHS and gut microbes have revealed a possible important link between the two. Animal experiments have shown that the development of VKHS may be associated with changes in the composition of gut microbes. Transplantation of gut microbes from patients with active VKHS into mice resulted in the observation of increased inflammatory symptoms in the uvea of these mice, suggesting that changes in gut microbes may play an important role in the pathogenesis of VKHS. The study suggests that treatment of VKHS patients with immunosuppressive agents not only controls inflammation but also restores the balance of the gut microbiota by modulating the gut microbial composition [73].

## 3 INDUCEMENT

Some studies have confirmed that certain vaccinations may be associated with an increased risk of autoimmune diseases, including ocular diseases, such as uveitis. Manni *et al*. [74] noted that patients developed VKH symptoms sometime after vaccination, primarily with severe acute respiratory syndrome coronavirus 2 and its coronavirus disease 2019. Bilateral serous retinal detachment with headache and vision loss after novel coronavirus inactivated vaccine has been reported; after systemic or topical steroid treatment, the serous retinal detachment disappeared, and vision improved [75, 76]. The adjuvant in the vaccine or some components of the vaccine may activate the body's immune response, thus triggering a series of clinical processes [75]. Rarely, ocular adverse events may result after vaccination with other types of vaccines, including hepatitis B [64], influenza [77], and BCG [78].

## 4 STAGING

Moorthy staging divides VKHS into (1) the prodromal phase, (2) the acute uveitis phase, (3) the recovery phase, and (4) the chronic recurrence phase based on its clinical features [79]. The division of VKHS into primary acute and chronic relapsing stages [80, 81], according to the progression of the disease, helps in early diagnosis and the development of individualized treatment and follow-up plans compared with the previous stages.

### 4.1 Primary Acute VKHS

The initial stage of the disease is characterized by prodromal symptoms; neurologic symptoms include headache, stiff neck, and mononucleosis in the cerebrospinal fluid, and auditory symptoms include mild vestibular syndrome and impaired high-frequency hearing [82]. Some patients also present with orbital pain, blurred vision, occasional photophobia, and tearing or skin sensory irritation in the head [83]. However, the above symptoms are not present in all patients [84, 85]; some patients have an acute onset and present with bilateral acute uveitis. Early manifestations are inflammation of the choroid alone, with chorioretinitis spreading to adjacent structures, optic disc, retina, and later progressively involving the anterior segment of the eye [86].

### 4.2 Recurrent Chronic VKHS

Chronic recurrent VKHS patients are mainly related to insufficient or inappropriate doses of immunotherapy. The treatment of chronic recurrent VKHS is a challenging task, especially in patients who respond poorly to treatment, are prone to complications, and are accompanied by more severe ocular and systemic symptoms and a more severe inflammatory response. Patients with recurrent chronic VKHS mainly present with late-onset fundus, Dalen-Fuchs nodules, and recurrent episodes of granulomatous anterior uveitis, with poor visual acuity and mean retinal sensitivity at late follow-up [87, 88]. A series of serious complications may occur during chronic recurrences of VKHS, such as glaucoma, cataracts, choroidal neovascularization, and lamellar choroidal retinal atrophy [83, 88, 89].

## 5 DIAGNOSIS

The diagnosis is based on its extraocular and ocular clinical signs and ancillary investigations, especially enhanced depth imaging optical coherence tomography (EDI-OCT), swept-source optical coherence tomography (SS-OCT), and indocyanine green angiography (ICGA) play an important role in the confirmation and treatment of VKHS [90, 91]. EDI-OCT, as well as SS-OCT, is a non-invasive imaging modality that is second only to ICGA in the evaluation of the choroid and allows monitoring of serous retinal detachment and choroidal thickness changes. It can be used to monitor subclinical choroidal inflammation during follow-up [92]. SS-OCT has two advantages over EDI-OCT; one is the high resolution, and the other is the number of images that can be measured, which allows for more accurate evaluation of the retina and choroid [93, 94]. ICGA is a non-invasive imaging technique that assesses the staging of the disease. It shows changes in choroidal perfusion and monitors the effectiveness of the treatment. It is the gold standard for confirming the diagnosis and following up this lesion [95, 96]. ICGA is able to clearly show the vascular structure and perfusion of the choroid [97, 98]. By looking at specific patterns in the ICGA images, it is possible to differentiate primary choroiditis, caused by VKHS, from secondary choroiditis due to other causes [99]. Moreover, ICGA helps to identify early signs of choroidal inflammation that may not be readily apparent on clinical examination, which opens up the possibility of early diagnosis and contributes to the development of more effective treatment plans [91, 100]. Choroidal thickness can be used as a follow-up indicator, with an increase in choroidal thickness in the early stages of recurrence and acute uveitis but a thinner choroidal thickness in patients recovering and with chronic disease [101]. At the same time, choroidal thickness is also an indicator of the degree of inflammation in patients with acute VKHS [102].

## 6 TREATMENT AND PROGNOSIS

The 2-4 weeks after the onset of the disease is considered to be a possible “window of opportunity” for the treatment of patients with VKHS [103, 104]. This window represents a time when the disease process is not fully developed and when treatment can prevent visual impairment due to chronic recurrence or complications [105]. Nonsteroidal immunomodulatory therapy, in combination with steroid therapy, has become the first-line treatment for VKHS. For patients with VKHS, especially those in the initial acute phase, the use of this combination therapy not only effectively controls the inflammatory response in the acute phase but also reduces the risk of chronic recurrent uveitis and decreases or prevents the incidence of serious complications, such as choroidal retinal atrophy and “late-onset fundus”, which ultimately contribute to an improved visual prognosis for patients [89, 106, 107]. A large body of clinical evidence suggests that single glucocorticoid therapy does not completely cure the disease and does not prevent its progression to chronicity [38, 84, 108]. In a 26-week trial comparing the efficacy of cyclosporine in combination with corticosteroids versus adalimumab in combination with corticosteroids for the treatment of VKHS, both combination therapies improved visual acuity and intraocular inflammatory response, but visual acuity improvement was improved by the former [109]. Novel biologic agents, such as anti-TNF-α agents, infliximab and adalimumab and anti-CD20 monoclonal antibodies, rituximab, have been shown to be effective in patients with refractory VKHS who are resistant to corticosteroids and immunosuppressive agents or who have had a poor response to treatment [110-112]. These biologic agents have extremely potent anti-inflammatory effects and significantly improve the visual prognosis of patients with refractory VKHS (Table **1**). However, biologic agents may activate the recurrence of tuberculosis, so it is essential that systemic tuberculosis screening should be done regularly during their use.

The prognosis of patients with VKHS depends on the duration of inflammation as well as the number and interval of relapses. Early high-dose long-term steroid therapy with early addition of first-line immuno-suppressive agents is essential to ensure improvement of optimal corrected visual acuity. During treatment, disease progression should be monitored using ICGA or OCT to facilitate a timely change of treatment regimen if subclinical chorioretinitis is detected.

## CONCLUSION

In summary, VKHS is a complex autoimmune disease whose pathogenesis involves factors at multiple levels. Current research has made significant progress in revealing the key pathophysiologic processes of this disease, and these advances are important for the development of more effective and safer therapies. A deeper understanding of the pathogenesis of VKHS will help to develop more precise and personalized treatment regimens and support the exploration of new therapeutic targets and approaches.

## Figures and Tables

**Table 1:** Mechanisms and disadvantages of the current main treatment modalities for Vogt-Koyanagi-Harada syndrome.

**Drugs**		**Mechanisms**	**Disadvantages**
Glucocorticosteroid		Powerful anti-inflammatory and immunosuppressive effects	Severe side effects and lack of tolerance
Immunosuppressive agents	Mycophenolate mofetil	Inhibits the activity of hypoxanthine nucleotides, thereby inhibiting the proliferation of T and B lymphocytes	Gastrointestinal upset is common, teratogenic effects and infections
	Cyclosporine A	A calmodulin phosphate inhibitor that inhibits T cell-mediated immunity and reduces the production of various pro-inflammatory cytokines	Hepatorenal toxicity, hypertension, metabolic abnormalities
	Cyclophosphamide	Belongs to the alkylating agent class of drugs that inhibit DNA synthesis by binding to DNA and also suppresses immune system function	Bone marrow suppression, hemorrhagic cystitis, reproductive toxicity, and teratogenicity
	Methotrexate	Antifolate metabolizers that interfere with cellular DNA and RNA synthesis and exert immunosuppressive and anti-inflammatory effects	Gastrointestinal discomfort, hematologic abnormalities, liver damage
	Azathioprine	Acts on purine metabolism during DNA synthesis and inhibits the activity of purine nucleotidase, thereby affecting the proliferation of immune-responsive cells and the function of immune cells	Bone marrow suppression, liver damage
Biologics	Adalimumab	Monoclonal antibodies against tumor necrosis factor-alpha (TNF-alpha), which attenuate inflammation and immune response by inhibiting the action of TNF-alpha.	Infection, injection site reaction. Active tuberculosis prohibited
	Infliximab		Upper respiratory tract infections, infusion-related reactions. Contraindicated in patients with tuberculosis or other active infections, infusion-related reactions, contraindicated in patients with tuberculosis or other active infections
	Rituximab	Inhibits B-cell proliferation and function by binding to the CD20 antigen on the surface of B-cells, thereby reducing inflammation and immune response	Infusion-related reactions, premedication with anti-allergy drugs prior to use
	Bevacizumab	Monoclonal antibody against vascular endothelial growth factor (VEGF), which controls ocular neovascularization and intraocular inflammatory response by inhibiting the action of VEGF	Bleeding, hypertension, proteinuria, thromboembolism
	Ranibizumab		Infection, intraocular inflammation, and increased intraocular pressure

## LIST OF ABBREVIATIONS

CCL1: C-C motif chemokine ligand 1
CMV: Cytomegalovirus
EDI-OCT: Enhanced depth imaging optical coherence tomography
FasL: Fas ligand
HLA: Human leukocyte antigen
IDO1: Indoleamine 2,3-dioxygenase 1
IL: Interleukin
MAPK: Mitogen-activated protease
miR-20a-5p: microRNA-20a-5p.
OSM: Oncostatin M
PBMCs: Peripheral blood mononuclear cells
PHGDH: Phosphoglycerate dehydrogenase 1
PI3K: Phosphatidylinositol 3-kinase
ROS: Reactive oxygen species
sEVs: Small extracellular vesicles
SS-OCT: Swept-source optical coherence tomography
Treg: T regulatory cells
TRIB1: Tribble pseudokinase 1
VKHS: Vogt-Koyanagi-Harada syndrome
